# The Role of the Cutaneous Microbiome in Hidradenitis Suppurativa—Light at the End of the Microbiological Tunnel

**DOI:** 10.3390/ijms21041205

**Published:** 2020-02-11

**Authors:** Ewan A. Langan, Andreas Recke, Therezia Bokor-Billmann, Franck Billmann, Birgit K. Kahle, Detlef Zillikens

**Affiliations:** 1 Department of Dermatology, University Medical Centre Schleswig-Holstein, Campus Luebeck, Ratzeburger Allee 160, 23538 Luebeck, Germany; andreas.recke@uksh.de (A.R.); birgit.kahle@uksh.de (B.K.K.); detlef.zillikens@uksh.de (D.Z.); 2 Dermatological Sciences, University of Manchester, Oxford Rd, Manchester M13 9PL, UK; 3 Department of Dermatology, University of Heidelberg, 69120 Heidelberg, Germany; Therezia.Bokor-Billmann@med.uni-heidelberg.de; 4 Department of Surgery, University of Heidelberg, 69120 Heidelberg, Germany; Franck.Billmann@med.uni-heidelberg.de

**Keywords:** acne inversa, hidradenitis suppurativa, microbiome, metagenomic

## Abstract

The development of next generation sequencing, coupled with advances in bio-informatics, has provided new insights into the role of the cutaneous microbiome in the pathophysiology of a range of inflammatory skin diseases. In fact, it has even been suggested that the identification of specific skin microbial signatures may not only be useful in terms of diagnosis of skin diseases but they may also ultimately help inform personalised treatment strategies. To date, research investigating the role of microbiota in the development of inflammatory skin diseases has largely focused on atopic eczema and psoriasis vulgaris. The role of the microbiome in Hidradenits suppurativa (HS)—also known as acne inversa—a chronic auto-inflammatory skin disease associated with significant morbidity, has received comparatively little attention. This is despite the fact that antimicrobial therapy plays a central role in the treatment of HS. After briefly outlining the clinical features of HS and current treatment strategies, we move on to review the evidence of microbial dysbiosis in HS pathophysiology. We conclude by outlining the potential for metagenomic studies to deepen our understanding of HS biology but more importantly to identify novel and much needed treatment strategies.

## 1. Background

Whilst the pathophysiology of HS remains to be clearly elucidated, the morbidity associated with the disease is profound, often under-estimated and may even exceed that encountered with other inflammatory skin diseases including psoriasis [1,2,3]. In addition to the psychosocial morbidity associated with HS, the economic costs of HS should not be underestimated [4]. In fact, in England alone, the average yearly hospital utilisation costs for patients with HS are over 2000 pounds per patient [5]. The economic costs are even more significant given that the patients are typically younger and of working age [6].

**Figure 1:**
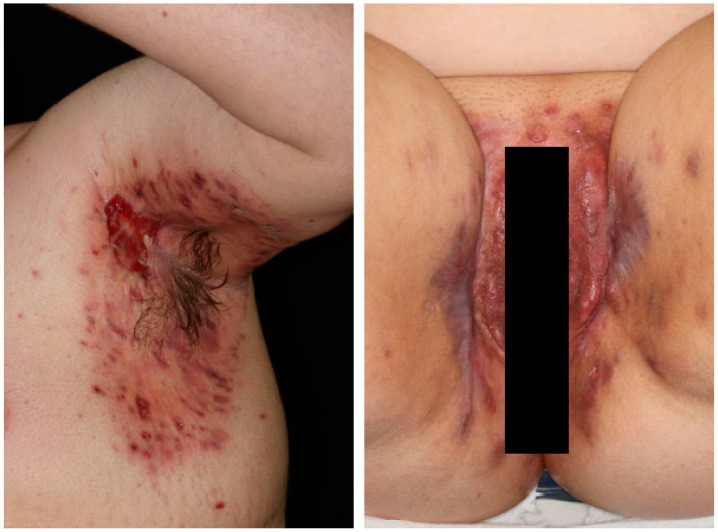
The clinical features of Hidradenitis suppurativa (HS). Erythema, erosions, pustules and scarring in the axilla of a male patient. Similar features in the perineal area in a female patient.

Clinically, the disease is hallmarked by the development of chronic and recurrent painful papules and nodules, abscesses, fistulae, sinus tracts and scarring in the typical predilection sites (axillae, inguinal fold, perianal and perineal regions, buttocks and infra/intermammary folds (Figure 1) [7]. Disease severity can be assessed using several measures, including the Hurley staging system, Sartorius scoring and the HS severity index. Whilst the precise incidence and prevalence of HS is unclear [8], due partly to the diagnostic difficulty associated with HS and the use of both registry data and self-report questionnaires [9]. Garg et al. reported a prevalence of 0.1% in a US-based population analysis, leading the authors to conclude that HS is an uncommon but not rare disease, with over-representation in young, female and African American patients [10]. The current multi-modal treatment approach includes the use of intralesional steroids, surgical and laser therapy, topical and systemic medical antimicrobial therapy and biologic treatments [11].

In terms of antimicrobial therapy, Mendonca et al. [12] reported the efficacy of a ten week combined course of rifampicin and clindamycin in small retrospective study of 14 patients, of which over half achieved a clinical remission. A number of subsequent, predominantly retrospective, studies have confirmed the efficacy of rifampicin and clindamycin in the management of HS but there has been no randomised control trial of this treatment regime to date [13,14,15,16]. Moreover, there remains a debate centred on treatment duration, long-term safety, the need for combined antimicrobial versus single agent therapy and the risk posed by the development of antibiotic resistance [17,18,19,20,21,22,23].

Based on the results of Phase III clinical trials, the anti-tumour necrosis factor alpha (anti-TNFα) monoclonal antibody adalimumab has also been added to the therapeutic armamentarium for moderate to severe HS [24]. Although Infliximab was the first biologic to demonstrate efficacy in the management of moderate to severe HS in a randomised, double-blind, placebo-controlled cross-over trial, its use remains off-label [25,26]. In a comprehensive review of the on-going or completed clinical trials for HS, Maarouf et al. [27] identified the following treatment targets—TNFα inhibition, the use of IL-1R antagonists, molecules targeting the IL-17 and IL-12/23 signalling pathways and complementary 5a inhibitors. More recently, the effect of the phosphodiesterase 4 inhibitor Apremilast has been examined in two studies, namely a phase II open label trial [28] and a small randomised placebo controlled trial [29]. Both studies demonstrated a clinical response associated with Apremilast and the treatment was generally well tolerated. Given the small sizes and treatment/follow-up periods (ranging from 16 to 24 weeks), further studies are required to determine the extent to which whether phosphodiesterase 4 inhibition will play a future role in the management of HS.

A combination of treatment modalities is often necessary to optimise outcomes [30]. Whilst this has traditionally focussed on the combination surgical and medical treatment strategies, recent studies have explored the effect of combining systemic medical treatments. For example, based on a retrospective analysis of the treatment of dermatologist diagnosed HS, treated in academic medical centres in the US, McPhie et al. [31] identified several drug combinations as potentially promising treatment strategies. These included retinoids and anti-androgens for mild disease, retinoids or tetracycline antibiotics and adalimumab for moderate disease and cyclosporine and adalimumab for severe disease. Whilst additional case reports highlight the successful treatment of moderate to severe HS with combined biologic treatments (adalimumab and ustekinumab) [32] and biological treatments combined with systemic steroid therapy [33], the current evidence base to support these strategies is limited. Another innovate approach is the combination of surgery (radical resection) with adjuvant biologic treatment [34]. DeFazio et al. [34] combined radical resection with post-operative infliximab or ustekinumab for an average of 10.5 months. New disease developed in 18% and 50% of the combined surgery and biologic treated versus surgical monotherapy patients respectively. Future clinical trials, specifically examining the effects of combined therapies are urgently needed to determine the efficacy and short- and long-term safety of these treatment approaches.

## 2. Characterising the Cutaneous Microbiota in HS

Despite the development of new therapeutic agents for the treatment of HS and established treatment algorithms, the clinical course of the disease is frequently chronic and often refractory to multiple treatment modalities [7,11]. Therefore, in order to develop new treatment strategies, research is currently focussed on developing a more comprehensive understanding of the pathophysiology of HS, in which the cutaneous microbiota may play an important role.

In order to review the evidence that HS is associated with changes in the cutaneous and gastrointestinal microbiomes, a PubMed based review of the English language literature was performed. Search terms include “Acne Inversa,” “Hidradentis Suppurativa,” “microbiome,” “microbiota,” “biologics,” “TNFα,” “IL-17,” “IL12/23,” “clinical trial,” and “antimicrobial peptides.” The PubMed data based was accessed between the 1. November 2019 and the 5. February 2020.

**Figure 2:**
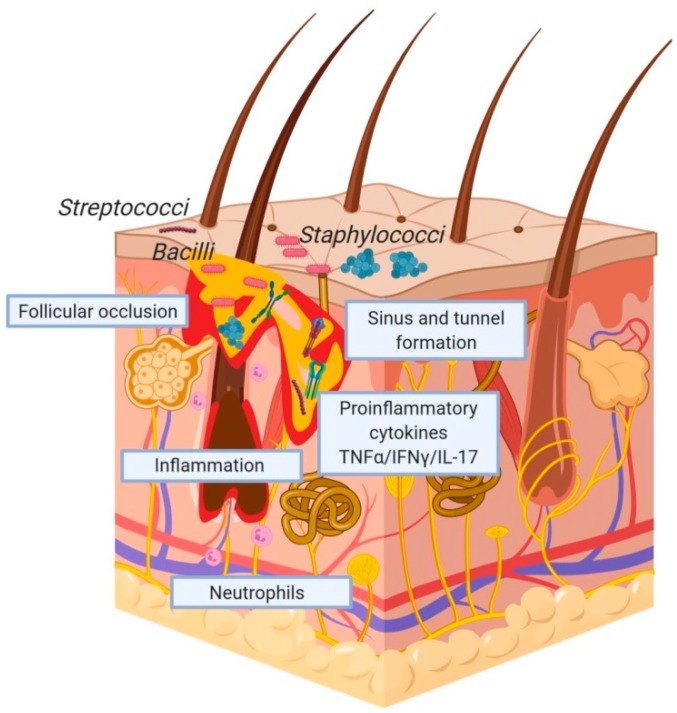
Hidradenitis suppurativa pathogenesis. Inflammation, follicular occlusion, neutrophilic infiltration and a pro-inflammatory cytokine milieu. Microbial colonisation is shown. It remains unclear whether the colonisation results from the disease or contributes to its pathophysiology. The figure was made in ©BioRender—biorender.com.

Indeed, there is increasing evidence that HS is associated with specific changes in the cutaneous microbiome (Figure 2) [9]. Traditionally, characterisation of the composition of the cutaneous microbiome in HS has relied heavily on direct bacterial culture using superficial swabs [35]. However, aspiration of pus has also been utilised to obtain samples for culture. For example, almost two decades ago, Brook et al. described the polymicrobial nature of axillary HS with *Staphylococcus aureus, Streptococcus pyogenes* and *Pseudomonas aeruginosa* the most prominent aerobic bacteria and *Pepto- streptococcus* spp., *Prevotella* spp., micro-aerophilic streptococci, *Fusobacterium* spp. and *Bacteroides* spp., the most common anaerobes [36]. Whilst this was a relatively small study, in which over one third of the patients had already received antibiotic therapy, it did identify the range of bacteria which could be cultured and highlighted the importance of anaerobic culture. More recently, in a prospective culture-based study of 46 patient with HS, Benzecry et al. [37] reported positive bacterial cultures in over half of the cases, with severe disease associated with an increased likelihood of positive bacterial culture. This led the authors to speculate that bacterial superinfection may play a role in the maintenance of inflammation.

Given that bacterial cultures may have simply represented colonisation of and/or contamination by resident or transient skin bacteria, subsequent efforts were undertaken to mitigate this problem by obtaining microbiological samples following CO_2_ laser ablation of disease tissue. Both superficial and deep level cultures following laser ablation were frequently positive, with *Staphylococcus aureus* and coagulase-negative staphylococci (CNS) the species most commonly identified. In terms of anaerobic bacteria, *Peptostreptococcus* sp. and *Propionibacterium acnes* were the most frequently identified [38]. The same group performed a similar study in acute HS flares in 10 patients and confirmed both the predominance of CNS at both the deep and superficial levels and the polymicrobial nature of the HS cutaneous flora [39]. Interestingly, *Staphylococcus aureus* was not present in the cultures from the acute lesions. Given the small sample size and lack of information regarding topical and/or systemic antibiotic use, the inability to culture *Staphylococcus aureus* should be interpreted with caution. Nevertheless, it does raise the possibility of both temporal and spatial changes in the cutaneous microbiome in HS which may be associated with disease flares. Of course, whether it causes or merely reflects disease activity remains unclear.

Moving on from the reliance on traditional bacterial culture, Ring et al. conducted a case-control study using peptide nucleic acid (PNA)-FISH probes in combination with confocal microscopy to determine the microbiota present in normal appearing skin in patients with HS compared to site-matched skin in healthy controls [40]. The study used skin biopsies rather than skin swabs or aspirates. Although the study was not sex-matched and the results were not validated by bacterial culture, fewer bacteria and reduced biofilm formation were seen in non-lesional HS skin when compared to that in healthy controls. Indeed, the hair follicles in the healthy control group were associated with marked biofilm formation which invited the authors to speculate that this may be protective. Moreover, the identification of biofilm formation sits well with the aforementioned studies demonstrating the preponderance of CNS in lesional HS skin.

Most recently, in a landmark review, Ring et al. confirmed that CNS, *Staphylococcus aureus* and mixed anaerobic bacteria are the most commonly identified bacteria in HS studies [41]. The authors review included six studies where bacterial identification was based on aerobic and anaerobic culture using swabs, skin biopsies or aspirates. Despite methodological differences in terms of specimen collection, disease-site location, antibiotic use and whether acute or chronic lesions were investigated, all of the studies identified members of the Firmicutes phylum (CNS and *Streptococci*), with *Staphylococcus aureus* present in over 80% of the studies [35,36,38,39,42,43]. In another review of the bacteriology of HS, Nikolakis et al. [44] also reported that 7 out of 9 studies reported the presence of anaerobic bacteria and otherwise a preponderance of CNS and *Staphylococcus aureus.* It should be noted that several of the studies were examined in both the reviews by Nikolakis and Ring et al. [41,44] leading to some overlap. Nonetheless, from the classical bacterial culture studies to date, HS is clearly associated with polymicrobial bacterial culture, with CNS, *Staphylococcus aureus* and anaerobes frequently identified irrespective of sampling method.

## 3. The benefits of 16S rRNA Sequencing and Metagenomic Approaches

Bearing in mind that traditional culture methods may only identify a tiny proportion of the cutaneous microbiome, perhaps even less than 1% [45], there has been a shift towards characterisation of cutaneous microbiota using 16S rRNA sequencing. Drawing on the techniques described in the seminal publications of Grice et al. [45,46,47], new insights have been gained into the bewildering complexity of individual cutaneous microbiomes. Not only is the cutaneous microbiome extremely diverse and highly individual but is also tightly regulated both within and between difference skin regions and stable over time. In fact, this stability over time means that individual microbial signatures or “fingerprints” have been the focus of research in forensic medicine, even allowing the identification of individuals based on the retrieval of the microbial information from surfaces the individual has touched [48].

In terms of cutaneous pathophysiology, shifts in the composition of the cutaneous microbiome (dysbiosis) have been identified in a range of inflammatory and autoimmune conditions including psoriasis [49,50,51,52], atopic eczema [53,54,55], bullous pemphigoid [56,57] and acne vulgaris [58]. Building on the evidence from culture-based studies that HS is associated with dysbiosis, Guet-Revillet et al. [59] employed both culture and metagenomic techniques to comprehensively characterise the cutaneous microbiome in HS. In the largest 16S rRNA based study of HS to date, 82 patients with HS were recruited and samples obtained via swabbing, aspiration and biopsy. Not only were the samples obtained from multiple sites but also from various stages of disease according to the Hurley classification. Patients who had undergone topical or systemic antibiotic treatment in the month prior to study participation were excluded. Based on bacterial culture, two microbiological profiles became apparent; profile A in which *Staphylococcus lugdunensis* predominated and profile B which was characterised by a mixed anaerobic flora but including anaerobic actinomycetes and/or *Streptococcus milleri*. Moreover, there was evidence that the microbiological profile varied with affected site and Hurley stage, for example profile A was associated with Hurley Stage 1 disease, particularly buttocks and mammary region, whilst profile B and C was more commonly associated with stage 2 and 3 disease [59]. Metagenomic sequencing, albeit from only 6 HS lesions, revealed *Staphylococcus* to be the predominant taxon in Hurley stage 1 and anaerobic species (*Prevotella, Porphyromonas, Anaerococcus and Mobiluncus* spp.) dominated in chronic suppurating lesions.

In order to more carefully define the anaerobic bacterial populations associated with HS, the same group performed a follow-up prospective metagenomic study in 65 patients with HS [60]. Furthermore, the study examined both lesional and non-lesional skin. In terms of bacterial culture, HS lesional skin was colonised by anaerobic bacteria and normal skin commensals. However, there were differences between lesional and non lesional skin. For example, *Streptococcus dysgalactiae* was only present in lesions, in which the proportion of commensal bacterial was significantly lower than in non lesional skin. Whilst alpha diversity was similar between lesional and non lesional skin, there was evidence of specific differences in the composition of the cutaneous microbiome according to lesional status. Namely, in non lesional skin, *Actinobacteria* and *Firmicutes* predominated, with the anaerobic microbiome rich in the genera *Anaerococcus*, *Finegoldia* and *Peptoniphilus*, members of the *Clostridiaceae* family. In contrast, lesional samples were hallmarked by an increased proportion of anaerobic gram-negative rods from the *Bacteroidetes* and *Fusobacteria* phyla, specifically of the *Prevotella, Porphyromonas and Fusobacterium* genera [60]. Moreover, the composition of the HS microbiota varied with clinical severity, with *Prevotella* and *Porphyromonas* associated with lesions. Consistent with the early metagenomic studies [59,60], recent studies have confirmed the association between *Prevotella* and *Porphyromonas* and HS, particularly in HS tunnels [61].

A limitation of the metagenomics studies to date has been the lack of a control group, particularly site-matched healthy controls. Several recent studies have specifically addressed this issue. Naik et al. [62] investigated the cutaneous microbiome at predefined predilection sites for HS in 12 subjects (predominantly Hurley stage 2) and 5 healthy controls. There was a decreased relative abundance of the commensal *Cutibacterium* spp. in HS compared to controls, whereas gram positive and negative anaerobes predominated in HS skin. Bacterial diversity was increased in HS skin when compared to healthy controls, at least in terms of the inguinal region. Consistent with Ring et al. [61], there was an abundance of *Prevotella* and *Porphyromonas*, which may underpin an association between these bacteria and disease severity. As the authors themselves acknowledged, the cross-sectional nature of the study and relatively small number of participants should be borne in mind before extrapolating the results to the wider HS population. However, it is also worth drawing attention to the use of the V1-V3 hypervariable region of the 16S ribosomal RNA gene for sequencing, given that the V1-V3 region may better capture the cutaneous microbiota populations when compared to the V4 region [63].

However, given that bacterial colonisation, especially in the inguinal region, may include typical members of the gastrointestinal microbiota, Schneider et al. [64] deliberated targeted the V3–V4 region to identify both the skin and gastrointestinal-derived skin microbiota in HS. Not only did the study include a healthy control population but in addition both lesional and non lesional HS was evaluated using swabs and glue-based follicular biopsies. Beta diversity was significantly decreased in both lesional and non lesional HS skin when compared to healthy controls. The authors were unable to confirm a difference in the bacterial composition of non lesional and lesional HS skin; a finding which had been reported in previous studies using bacterial culture, nucleic acid fluorescence in situ hybridization and metagenomics [40,60]. However, it should be noted that there were significant methodological differences between the studies, especially in terms of sampling technique, which may have influenced the results. Indeed, the optimal sampling technique for cutaneous microbiome studies remains an area of debate [45,65,66].

## 4. Environmental Factors

Any examination of the cutaneous microbiome in health and disease must take account of environmental factors. In terms of HS etiopathology, the association between smoking and HS is well known and established. Whilst a recent meta-analysis of 25 studies (101,977 HS patients and 17,194,921 non-HS controls) was unable to demonstrate a causal relationship between smoking and the development of HS, patients with HS were four times more likely to smoke [67]. To date, no studies have specifically addressed the effect of smoking on the cutaneous microbiome in general and on the cutaneous microbiome in HS in particular.

However, there is evidence that tobacco smoke effects both the pulmonary [68] and, perhaps more interestingly, the gastrointestinal microbiome [69]. Smoking is not only associated with a decreased diversity in the composition of the gastrointestinal microbiome but also with promoting specific bacterial genera, including Bacteroides, Prevotella, Enterobacteria and Clostridium [69]. Given the potential interaction between the gastrointestinal and cutaneous microbiomes, it is at least conceivable that tobacco smoke may directly and/or indirectly affect the cutaneous microbiome, potentially contributing to the development of HS. This intriguing possibility underpinned by research demonstrating the altered gastrointestinal microbiome in patients with inflammatory dermatoses [70]. Indeed, there is a growing interest in the gut-skin-axis, with probiotics already being postulated as a future treatment strategy for skin disorders including HS [71].

There is clearly an urgent need to definitely characterise both the cutaneous and gastrointestinal microbiome in HS, not only given the association between HS and inflammatory bowel disease [72] but also given the role of antimicrobial therapy in the management of HS, which itself can dramatically and fundamentally alter the composition of the gastrointestinal bacterial flora. The extent to which these changes may contribute to the HS pathophysiology, either promoting disease progression or even serving a protective role, needs to be urgently clarified to optimise treatment selection. It remains to be seen whether the composition of the gastrointestinal microbiome is a risk factor for the development of HS, dependent on additional genetic and environmental factors and whether it influences treatment success with antimicrobial and/or biologics, analogous to the effect of checkpoint inhibitors in the treatment of metastatic melanoma [73].

## 5. Limitations of the Current Metagenomic Evidence Base

Perhaps the most striking methodological concerns with the culture-based and metagenomic studies in HS to date are the reliance on cross-sectional study designs and sampling at single time points. These concerns are compounded by the use of various sampling techniques and a wide spectrum of sampling sites [61]. It is readily accepted that the cutaneous microbiome is both skin-site and skin-microenvironment dependent and may be influenced by the use of topical antiseptic agents, albeit in the short-term [74]. Moreover, systemic tetracycline antibiotics and retinoids have also been shown to affect the composition of the cutaneous microbiome in acne vulgaris [75]. These latter factors are of particular relevance given that local antiseptic and systemic antibiotic treatment are part of the mainstay of clinical management of HS [76].

Therefore, in order to address these concerns, standardised, prospective, longitudinal studies of the cutaneous microbiome in HS are urgently required, ideally with samples from involved and non-involved skin and including a control group. Not only would such studies potentially shed new light on the pathophysiology of the disease but they may also identify novel biomarkers of disease activity and treatment response. In fact, given that HS is a chronic inflammatory disease, which frequently undergoes periods of relapse and remission, longitudinal non-invasive metagenomic studies could identify any changes in the composition of cutaneous microbiome over time. If these changes were correlated to disease flares, this would open up the possibly of adjusting and/or intensifying treatment in this “prodromal phase” to prevent exacerbations.

Indeed, metagenomic studies in psoriasis and atopic dermatitis have blazed a trail in this respect. For example, there is evidence of temporal shifts in the composition of the cutaneous microbiome in patients with atopic dermatitis related to both treatment and disease flares [53,77]. Furthermore, systemic treatment has been shown to affect the cutaneous microbiome in patients with psoriasis, increasing the ratio of actinobacteria to firmicutes [49,78]. In fact, there is evidence that combining metagenomic sequencing based studies with traditional bacterial culture and identification with mass spectrometry may shed additional light on composition and ultimately function, of cutaneous microbiota [78]. Of course, association studies cannot ultimately determine whether changes in the cutaneous microbiome contribute to the disease pathophysiology or result from it. Nevertheless, longitudinal studies of both lesional and non-lesional skin in patients with HS can potentially identify dysbiosis associated with the transition from non-lesional to lesional skin; providing a potential therapeutic target.

## 6. The Cutaneous and/or Gastrointestinal Microbiomes as a Therapeutic Targets

In terms of HS therapy, biologic agents, particularly targeting tumour necrosis factor alpha (TNFα), are now an integral part of the management of moderate to severe disease [79,80,81]. Although clinical benefit has been reported with several biologics, including infliximab [82], ustekinumab [83] and secukinumab [84], only adalimumab is licensed at present for the treatment of moderate to severe HS. Bearing in mind that the use of biologics is associated with changes in the composition of both the cutaneous [78,85] and gastrointestinal microbiomes [86], it is at least conceivable that biomarkers of treatment response may be identified in future metagenomic studies. Moreover, there is emerging evidence that chronic inflammatory skin disorders may be associated with specific perturbations in the gastrointestinal microbiome. For example, whilst psoriasis was associated with reduced levels of *Faecalibacterium prausnitzii* and increased levels of *Escherichia coli*, this was not the case in HS [70]. The cross-talk between the cutaneous and gut microbiome, termed the gut-skin axis, is currently the focus of intense research [87]. Indeed, there is evidence that exposure of the skin to ultraviolet radiation can alter the gut microbiome [88] and modulation of the gut microbiome may represent a novel mechanism to treat skin diseases [71], although prospective randomized clinical trials are lacking.

Understanding the complex interplay between the cutaneous and gastrointestinal microbiome, which may be largely regulated by both the innate and acquired immune systems, is one of the central challenges facing metagenomic research. There is an urgent need to comprehensively map the skin-site-specific and disease-dependent composition of the cutaneous microbiome in HS in order to generate new insights into the pathophysiology of HS and ultimately identify novel treatment strategies and targets. It is also important to re-examine our understanding of the skin microbiota as passive bystanders in an immune-driven autoinflammatory disease and to explore the possibility that the bacteria themselves may play a role a significant role in disease flares or even serve as protective factors.

In this regard, it is interesting to note that HS is associated with the expression of cutaneous anti-microbial peptides including cathelicidin in the apocrine sweat gland and distal outer root sheath of the hair follicle (HF) epithelium in lesional skin. In addition to cathelicidin, the expression of psoriasin, human β-defensin 3, α-melanocyte stimulating hormone, macrophage migration inhibitory factor and tumor necrosis factor are all significantly increased in HS epidermis when compared to healthy controls [89]. Additional cytokines, including IL-17, IL-1β and interferon γ have also been implicated in the pathogenesis of HS [90,91]. Future metagenomic research would be well advised to investigate the potential mechanisms through which bacteria, both resident and transient, can modulate the local inflammatory milieu to contribute to or even prevent disease flares.

## 7. Conclusions

Hidradenitis suppurativa is a chronic, debilitating inflammatory skin disease whose pathophysiology remains poorly understood [92]. Although the disease itself is relatively uncommon, it is frequently associated with significant physical and psychological morbidity [93] and undoubtedly represents an area of unmet clinical need [2]. Whilst it has long been recognized that HS is associated with changes in the resident bacterial flora, only now are metagenomic studies starting to shed new light on the diversity, complexity and regulation of the cutaneous microbiome in HS. Ultimately, prospective, controlled, longitudinal studies of the microbiome in HS lesional and non lesional skin are required, potentially complemented by genetic studies [94,95], to comprehensively determine the extent to which genetic and environmental factor contribute to dysbiosis and consequently disease pathology, but, more importantly, to identify much needed novel therapeutic targets.

## References

[1] Kolli S.S., Senthilnathan A., Cardwell L.A., Richardson I.M., Feldman S.R., Pichardo R.O. (2019). Hidradenitis suppurativa has an enormous impact on patients’ lives. J. Am. Acad. Dermatol..

[2] Garg A., Neuren E., Cha D., Kirby J.S., Ingram J.R., Jemec G.B.E., Esmann S., Thorlacius L., Villumsen B., Marmol V.D. (2019). Evaluating Patients’ Unmet Needs in Hidradenitis Suppurativa: results from the Global VOICE project. J. Am. Acad. Dermatol..

[3] Keary E., Hevey D., Tobin A.M. (2019). A qualitative analysis of psychological distress in hidradenitis suppurativa. Br. J. Dermatol..

[4] Marvel J., Vlahiotis A., Sainski-Nguyen A., Willson T., Kimball A. (2019). Disease burden and cost of hidradenitis suppurativa: a retrospective examination of US administrative claims data. BMJ Open.

[5] Desai N., Shah P. (2017). High burden of hospital resource utilization in patients with hidradenitis suppurativa in England: a retrospective cohort study using hospital episode statistics. Br. J. Dermatol..

[6] Kirby J.S., Miller J.J., Adams D.R., Leslie D. (2014). Health care utilization patterns and costs for patients with hidradenitis suppurativa. JAMA Dermatol..

[7] Vekic D.A., Frew J., Cains G.D. (2018). Hidradenitis suppurativa, a review of pathogenesis, associations and management. Part 1. Australas J. Dermatol..

[8] Bettoli V., Cazzaniga S., Scuderi V., Zedde P., Di Landro A., Naldi L., Group I. (2019). Hidradenitis suppurativa epidemiology: from the first Italian registry in 2009 to the most recent epidemiology updates—Italian Registry Hidradenitis Suppurativa project 2. J. Eur. Acad. Dermatol. Venereol..

[9] Goldburg S.R., Strober B.E., Payette M.J. (2019). Part I. Hidradenitis Suppurativa: Epidemiology, clinical presentation and pathogenesis. J. Am. Acad. Dermatol..

[10] Garg A., Kirby J.S., Lavian J., Lin G., Strunk A. (2017). Sex- and Age-Adjusted Population Analysis of Prevalence Estimates for Hidradenitis Suppurativa in the United States. JAMA Dermatol..

[11] Vekic D.A., Cains G.D. (2018). Hidradenitis suppurativa, a review of pathogenesis, associations and management. Part 2. Australas J. Dermatol..

[12] Mendonca C.O., Griffiths C.E. (2006). Clindamycin and rifampicin combination therapy for hidradenitis suppurativa. Br. J. Dermatol..

[13] Bettoli V., Zauli S., Borghi A., Toni G., Minghetti S., Ricci M., Virgili A. (2014). Oral clindamycin and rifampicin in the treatment of hidradenitis suppurativa-acne inversa: a prospective study on 23 patients. J. Eur. Acad. Dermatol. Venereol..

[14] Dessinioti C., Zisimou C., Tzanetakou V., Stratigos A., Antoniou C. (2016). Oral clindamycin and rifampicin combination therapy for hidradenitis suppurativa: a prospective study and 1-year follow-up. Clin. Exp. Dermatol..

[15] Gener G., Canoui-Poitrine F., Revuz J.E., Faye O., Poli F., Gabison G., Pouget F., Viallette C., Wolkenstein P., Bastuji-Garin S. (2009). Combination therapy with clindamycin and rifampicin for hidradenitis suppurativa: a series of 116 consecutive patients. Dermatology.

[16] van der Zee H.H., Boer J., Prens E.P., Jemec G.B. (2009). The effect of combined treatment with oral clindamycin and oral rifampicin in patients with hidradenitis suppurativa. Dermatology.

[17] Albrecht J., Baine P.A., Ladizinski B., Jemec G.B., Bigby M. (2019). Long-term clinical safety of clindamycin and rifampicin combination for the treatment of hidradenitis suppurativa. A Critically Appraised Topic. Br. J. Dermatol..

[18] Marasca C., Masara A., Annunziata M.C., Bettoli V., Luciano M.A., Fabbrocini G. (2019). Long-term clinical safety of clindamycin and rifampicin combination for the treatment of hidradenitis suppurativa: a strategy to reduce side-effects, improving patients’ compliance. Br. J. Dermatol..

[19] Albrecht J., Barbaric J., Nast A. (2019). Rifampicin alone may be enough: is it time to abandon the classic oral clindamycin-rifampicin combination for hidradenitis suppurativa?. Br. J. Dermatol..

[20] Hambly R., Kirby B. (2019). Prolonged clindamycin and rifampicin for hidradenitis suppurativa: resist to prevent resistance. Br. J. Dermatol..

[21] Sayed C., Alikhan A. (2019). Response to “Rifampin and Clindamycin are safe long term”. J. Am. Acad. Dermatol..

[22] Schneller-Pavelescu L., Vergara-de Caso E., Martorell A., Romani J., Lazaro M., Vilarrasa E., Diaz-Ley B., Vazquez-Osorio I., Segura Palacios J.M., Azana J.M. (2019). Reply to “Comment on ‘Interruption of oral clindamycin plus rifampicin therapy in patients with hidradenitis suppurativa: an observational study to assess prevalence and causes’ ”. J. Am. Acad Dermatol.

[23] Albrecht J., Bigby M. (2019). Rifampin and Clindamycin are safe long term Response to North American clinical management guidelines for hidradenitis suppurativa: A publication from the United States and Canadian Hidradenitis Suppurativa Foundations Part II: Topical, intralesional and systemic medical management. J. Am. Acad Dermatol.

[24] Kimball A.B., Okun M.M., Williams D.A., Gottlieb A.B., Papp K.A., Zouboulis C.C., Armstrong A.W., Kerdel F., Gold M.H., Forman S.B. (2016). Two Phase 3 Trials of Adalimumab for Hidradenitis Suppurativa. N Engl. J. Med..

[25] Sand F.L., Thomsen S.F. (2015). Off-label use of TNF-alpha inhibitors in a dermatological university department: retrospective evaluation of 118 patients. Dermatol. Ther..

[26] Grant A., Gonzalez T., Montgomery M.O., Cardenas V., Kerdel F.A. (2010). Infliximab therapy for patients with moderate to severe hidradenitis suppurativa: a randomized, double-blind, placebo-controlled crossover trial. J. Am. Acad. Dermatol..

[27] Maarouf M., Clark A.K., Lee D.E., Shi V.Y. (2018). Targeted treatments for hidradenitis suppurativa: a review of the current literature and ongoing clinical trials. J. Dermatolog. Treat..

[28] Kerdel F.R., Azevedo F.A., Kerdel Don C., Don F.A., Fabbrocini G., Kerdel F.A. (2019). Apremilast for the Treatment of Mild-to-Moderate Hidradenitis Suppurativa in a Prospective, Open-Label, Phase 2 Study. J. Drugs Dermatol..

[29] Vossen A., van Doorn M.B.A., van der Zee H.H., Prens E.P. (2019). Apremilast for moderate hidradenitis suppurativa: Results of a randomized controlled trial. J. Am. Acad. Dermatol..

[30] Alikhan A., Sayed C., Alavi A., Alhusayen R., Brassard A., Burkhart C., Crowell K., Eisen D.B., Gottlieb A.B., Hamzavi I. (2019). North American clinical management guidelines for hidradenitis suppurativa: A publication from the United States and Canadian Hidradenitis Suppurativa Foundations: Part II: Topical, intralesional and systemic medical management. J. Am. Acad. Dermatol..

[31] McPhie M.L., Bridgman A.C., Kirchhof M.G. (2019). Combination Therapies for Hidradenitis Suppurativa: A Retrospective Chart Review of 31 Patients. J. Cutan. Med. Surg..

[32] Cline A., Pichardo R.O. (2019). Successful treatment of hidradenitis suppurativa in the setting of Crohn disease with combination adalimumab and ustekinumab. Dermatol. Online J..

[33] Molina-Leyva A. (2019). Adalimumab every other week combined with dexamethasone pulses for the treatment of refractory hidradenitis suppurativa. Dermatol. Ther..

[34] DeFazio M.V., Economides J.M., King K.S., Han K.D., Shanmugam V.K., Attinger C.E., Evans K.K. (2016). Outcomes After Combined Radical Resection and Targeted Biologic Therapy for the Management of Recalcitrant Hidradenitis Suppurativa. Ann. Plast Surg..

[35] Highet A.S., Warren R.E., Staughton R.C., Roberts S.O. (1980). Streptococcus milleri causing treatable infection in perineal hidradenitis suppurativa. Br. J. Dermatol..

[36] Brook I., Frazier E.H. (1999). Aerobic and anaerobic microbiology of axillary hidradenitis suppurativa. J. Med. Microbiol..

[37] Benzecry V., Grancini A., Guanziroli E., Nazzaro G., Barbareschi M., Marzano A.V., Muratori S., Veraldi S. (2018). Hidradenitis suppurativa/acne inversa: a prospective bacteriological study of 46 patients and review of the literature. G Ital. Dermatol. Venereol..

[38] Lapins J., Jarstrand C., Emtestam L. (1999). Coagulase-negative staphylococci are the most common bacteria found in cultures from the deep portions of hidradenitis suppurativa lesions, as obtained by carbon dioxide laser surgery. Br. J. Dermatol..

[39] Sartorius K., Killasli H., Oprica C., Sullivan A., Lapins J. (2012). Bacteriology of hidradenitis suppurativa exacerbations and deep tissue cultures obtained during carbon dioxide laser treatment. Br. J. Dermatol..

[40] Ring H.C., Bay L., Kallenbach K., Miller I.M., Prens E., Saunte D.M., Bjarnsholt T., Jemec G.B. (2017). Normal Skin Microbiota is Altered in Pre-clinical Hidradenitis Suppurativa. Acta Derm Venereol..

[41] Ring H.C., Riis Mikkelsen P., Miller I.M., Jenssen H., Fuursted K., Saunte D.M., Jemec G.B. (2015). The bacteriology of hidradenitis suppurativa: a systematic review. Exp. Dermatol..

[42] Jemec G.B., Faber M., Gutschik E., Wendelboe P. (1996). The bacteriology of hidradenitis suppurativa. Dermatology.

[43] Matusiak L., Bieniek A., Szepietowski J.C. (2014). Bacteriology of hidradenitis suppurativa - which antibiotics are the treatment of choice?. Acta Derm. Venereol..

[44] Nikolakis G., Join-Lambert O., Karagiannidis I., Guet-Revillet H., Zouboulis C.C., Nassif A. (2015). Bacteriology of hidradenitis suppurativa/acne inversa: A review. J. Am. Acad. Dermatol..

[45] Grice E.A., Kong H.H., Renaud G., Young A.C., Program N.C.S., Bouffard G.G., Blakesley R.W., Wolfsberg T.G., Turner M.L., Segre J.A. (2008). A diversity profile of the human skin microbiota. Genome Res..

[46] Grice E.A., Kong H.H., Conlan S., Deming C.B., Davis J., Young A.C., Program N.C.S., Bouffard G.G., Blakesley R.W., Murray P.R. (2009). Topographical and temporal diversity of the human skin microbiome. Science.

[47] Grogan M.D., Bartow-McKenney C., Flowers L., Knight S.A.B., Uberoi A., Grice E.A. (2019). Research Techniques Made Simple: Profiling the Skin Microbiota. J. Invest. Dermatol..

[48] Oliveira M., Amorim A. (2018). Microbial forensics: new breakthroughs and future prospects. Appl. Microbiol. Biotechnol..

[49] Alekseyenko A.V., Perez-Perez G.I., De Souza A., Strober B., Gao Z., Bihan M., Li K., Methe B.A., Blaser M.J. (2013). Community differentiation of the cutaneous microbiota in psoriasis. Microbiome.

[50] Fry L., Baker B.S., Powles A.V., Fahlen A., Engstrand L. (2013). Is chronic plaque psoriasis triggered by microbiota in the skin?. Br. J. Dermatol..

[51] Gao Z., Tseng C.H., Strober B.E., Pei Z., Blaser M.J. (2008). Substantial alterations of the cutaneous bacterial biota in psoriatic lesions. PLoS ONE.

[52] Tobin A.M. (2019). Unravelling the microbiome in psoriasis. Br. J. Dermatol..

[53] Kong H.H., Oh J., Deming C., Conlan S., Grice E.A., Beatson M.A., Nomicos E., Polley E.C., Komarow H.D., Program N.C.S. (2012). Temporal shifts in the skin microbiome associated with disease flares and treatment in children with atopic dermatitis. Genome Res..

[54] Paller A.S., Kong H.H., Seed P., Naik S., Scharschmidt T.C., Gallo R.L., Luger T., Irvine A.D. (2019). The microbiome in patients with atopic dermatitis. J. Allergy Clin. Immunol..

[55] Fyhrquist N., Muirhead G., Prast-Nielsen S., Jeanmougin M., Olah P., Skoog T., Jules-Clement G., Feld M., Barrientos-Somarribas M., Sinkko H. (2019). Microbe-host interplay in atopic dermatitis and psoriasis. Nat. Commun..

[56] Miodovnik M., Kunstner A., Langan E.A., Zillikens D., Glaser R., Sprecher E., Baines J.F., Schmidt E., Ibrahim S.M. (2017). A distinct cutaneous microbiota profile in autoimmune bullous disease patients. Exp. Dermatol..

[57] Scaglione G.L., Fania L., De Paolis E., De Bonis M., Mazzanti C., Di Zenzo G., Lechiancole S., Messinese S., Capoluongo E. (2019). Evaluation of cutaneous, oral and intestinal microbiota in patients affected by pemphigus and bullous pemphigoid: A pilot study. Exp. Mol. Pathol..

[58] Ramasamy S., Barnard E., Dawson T.L., Li H. (2019). The role of the skin microbiota in acne pathophysiology. Br. J. Dermatol..

[59] Guet-Revillet H., Coignard-Biehler H., Jais J.P., Quesne G., Frapy E., Poiree S., Le Guern A.S., Le Fleche-Mateos A., Hovnanian A., Consigny P.H. (2014). Bacterial pathogens associated with hidradenitis suppurativa, France. Emerg Infect. Dis.

[60] Guet-Revillet H., Jais J.P., Ungeheuer M.N., Coignard-Biehler H., Duchatelet S., Delage M., Lam T., Hovnanian A., Lortholary O., Nassif X. (2017). The Microbiological Landscape of Anaerobic Infections in Hidradenitis Suppurativa: A Prospective Metagenomic Study. Clin. Infect. Dis..

[61] Ring H.C., Sigsgaard V., Thorsen J., Fuursted K., Fabricius S., Saunte D.M., Jemec G.B. (2019). The microbiome of tunnels in hidradenitis suppurativa patients. J. Eur. Acad Dermatol. Venereol..

[62] Naik H.B., Jo J.H., Paul M., Kong H.H. (2019). Skin microbiota perturbations are distinct and disease severity-dependent in hidradenitis suppurativa. J. Invest. Dermatol..

[63] Meisel J.S., Hannigan G.D., Tyldsley A.S., SanMiguel A.J., Hodkinson B.P., Zheng Q., Grice E.A. (2016). Skin Microbiome Surveys Are Strongly Influenced by Experimental Design. J. Invest. Dermatol..

[64] Schneider A.M., Cook L.C., Zhan X., Banerjee K., Cong Z., Imamura-Kawasawa Y., Gettle S.L., Longenecker A.L., Kirby J.S., Nelson A.M. (2019). Loss of skin microbial diversity and alteration of bacterial metabolic function in Hidradenitis Suppurativa. J. Invest. Dermatol..

[65] Fahlen A., Engstrand L., Baker B.S., Powles A., Fry L. (2012). Comparison of bacterial microbiota in skin biopsies from normal and psoriatic skin. Arch. Dermatol. Res..

[66] Prast-Nielsen S., Tobin A.M., Adamzik K., Powles A., Hugerth L.W., Sweeney C., Kirby B., Engstrand L., Fry L. (2019). Investigation of the skin microbiome: swabs vs. biopsies. Br. J. Dermatol..

[67] Acharya P., Mathur M. (2019). Hidradenitis suppurativa and smoking: a systematic review and meta-analysis. J. Am. Acad. Dermatol..

[68] Yu G., Gail M.H., Consonni D., Carugno M., Humphrys M., Pesatori A.C., Caporaso N.E., Goedert J.J., Ravel J., Landi M.T. (2016). Characterizing human lung tissue microbiota and its relationship to epidemiological and clinical features. Genome Biol..

[69] Savin Z., Kivity S., Yonath H., Yehuda S. (2018). Smoking and the intestinal microbiome. Arch. Microbiol..

[70] Eppinga H., Sperna Weiland C.J., Thio H.B., van der Woude C.J., Nijsten T.E., Peppelenbosch M.P., Konstantinov S.R. (2016). Similar Depletion of Protective Faecalibacterium prausnitzii in Psoriasis and Inflammatory Bowel Disease but not in Hidradenitis Suppurativa. J. Crohns Colitis.

[71] Szanto M., Dozsa A., Antal D., Szabo K., Kemeny L., Bai P. (2019). Targeting the gut-skin axis-Probiotics as new tools for skin disorder management?. Exp. Dermatol..

[72] Phan K., Tatian A., Woods J., Cains G., Frew J.W. (2019). Prevalence of inflammatory bowel disease (IBD) in hidradenitis suppurativa (HS): systematic review and adjusted meta-analysis. Int. J. Dermatol..

[73] Gopalakrishnan V., Spencer C.N., Nezi L., Reuben A., Andrews M.C., Karpinets T.V., Prieto P.A., Vicente D., Hoffman K., Wei S.C. (2018). Gut microbiome modulates response to anti-PD-1 immunotherapy in melanoma patients. Science.

[74] SanMiguel A.J., Meisel J.S., Horwinski J., Zheng Q., Bradley C.W., Grice E.A. (2018). Antiseptic Agents Elicit Short-Term, Personalized and Body Site-Specific Shifts in Resident Skin Bacterial Communities. J. Invest. Dermatol..

[75] Kelhala H.L., Aho V.T.E., Fyhrquist N., Pereira P.A.B., Kubin M.E., Paulin L., Palatsi R., Auvinen P., Tasanen K., Lauerma A. (2018). Isotretinoin and lymecycline treatments modify the skin microbiota in acne. Exp. Dermatol..

[76] Hendricks A.J., Hsiao J.L., Lowes M.A., Shi V.Y. (2019). A Comparison of International Management Guidelines for Hidradenitis Suppurativa. Dermatology.

[77] Chng K.R., Tay A.S., Li C., Ng A.H., Wang J., Suri B.K., Matta S.A., McGovern N., Janela B., Wong X.F. (2016). Whole metagenome profiling reveals skin microbiome-dependent susceptibility to atopic dermatitis flare. Nat. Microbiol..

[78] Langan E.A., Kunstner A., Miodovnik M., Zillikens D., Thaci D., Baines J.F., Ibrahim S.M., Solbach W., Knobloch J.K. (2019). Combined culture and metagenomic analyses reveal significant shifts in the composition of the cutaneous microbiome in psoriasis. Br. J. Dermatol..

[79] Flood K.S., Porter M.L., Kimball A.B. (2019). Biologic Treatment for Hidradenitis Suppurativa. Am. J. Clin. Dermatol..

[80] Lee R.A., Eisen D.B. (2015). Treatment of hidradenitis suppurativa with biologic medications. J. Am. Acad. Dermatol..

[81] Ingram J.R., Collier F., Brown D., Burton T., Burton J., Chin M.F., Desai N., Goodacre T.E.E., Piguet V., Pink A.E. (2019). British Association of Dermatologists guidelines for the management of hidradenitis suppurativa (acne inversa) 2018. Br. J. Dermatol..

[82] Ghias M.H., Johnston A.D., Kutner A.J., Micheletti R.G., Hosgood H.D., Cohen S.R. (2019). High-dose, high-frequency infliximab: A novel treatment paradigm for hidradenitis suppurativa. J. Am. Acad. Dermatol..

[83] Takeda K., Kikuchi K., Kanazawa Y., Yamasaki K., Aiba S. (2019). Ustekinumab treatment for hidradenitis suppurativa. J. Dermatol..

[84] Prussick L., Rothstein B., Joshipura D., Saraiya A., Turkowski Y., Abdat R., Alomran A., Zancanaro P., Kachuk C., Dumont N. (2019). Open-label, investigator-initiated, single-site exploratory trial evaluating secukinumab, an anti-interleukin-17A monoclonal antibody, for patients with moderate-to-severe hidradenitis suppurativa. Br. J. Dermatol..

[85] Loesche M.A., Farahi K., Capone K., Fakharzadeh S., Blauvelt A., Duffin K.C., DePrimo S.E., Munoz-Elias E.J., Brodmerkel C., Dasgupta B. (2018). Longitudinal Study of the Psoriasis-Associated Skin Microbiome during Therapy with Ustekinumab in a Randomized Phase 3b Clinical Trial. J. Invest. Dermatol..

[86] Yeh N.L., Hsu C.Y., Tsai T.F., Chiu H.Y. (2019). Gut Microbiome in Psoriasis is Perturbed Differently During Secukinumab and Ustekinumab Therapy and Associated with Response to Treatment. Clin. Drug Investig..

[87] Salem I., Ramser A., Isham N., Ghannoum M.A. (2018). The Gut Microbiome as a Major Regulator of the Gut-Skin Axis. Front. Microbiol..

[88] Bosman E.S., Albert A.Y., Lui H., Dutz J.P., Vallance B.A. (2019). Skin Exposure to Narrow Band Ultraviolet (UVB) Light Modulates the Human Intestinal Microbiome. Front. Microbiol..

[89] Emelianov V.U., Bechara F.G., Glaser R., Langan E.A., Taungjaruwinai W.M., Schroder J.M., Meyer K.C., Paus R. (2012). Immunohistological pointers to a possible role for excessive cathelicidin (LL-37) expression by apocrine sweat glands in the pathogenesis of hidradenitis suppurativa/acne inversa. Br. J. Dermatol..

[90] Kelly G., Hughes R., McGarry T., van den Born M., Adamzik K., Fitzgerald R., Lawlor C., Tobin A.M., Sweeney C.M., Kirby B. (2015). Dysregulated cytokine expression in lesional and nonlesional skin in hidradenitis suppurativa. Br. J. Dermatol..

[91] Vossen A., van der Zee H.H., Prens E.P. (2018). Hidradenitis Suppurativa: A Systematic Review Integrating Inflammatory Pathways Into a Cohesive Pathogenic Model. Front. Immunol..

[92] Thorlacius L., Cohen A.D., Gislason G.H., Jemec G.B.E., Egeberg A. (2018). Increased Suicide Risk in Patients with Hidradenitis Suppurativa. J. Invest. Dermatol..

[93] Machado M.O., Stergiopoulos V., Maes M., Kurdyak P.A., Lin P.Y., Wang L.J., Shyu Y.C., Firth J., Koyanagi A., Solmi M. (2019). Depression and Anxiety in Adults With Hidradenitis Suppurativa: A Systematic Review and Meta-analysis. JAMA Dermatol..

[94] Frew J.W., Navrazhina K., Byrd A.S., Garg A., Ingram J.R., Kirby J.S., Lowes M.A., Naik H., Piguet V., Prens E.P. (2019). Defining lesional, perilesional and unaffected skin in hidradenitis suppurativa: proposed recommendations for clinical trials and translational research studies. Br. J. Dermatol..

[95] Alavi A., Piguet V. (2019). Genotype-phenotype correlation in inherited hidradenitis suppurativa: one step forward, one step back. Br. J. Dermatol..

